# Validation of a Wearable Sensor Prototype for Measuring Heart Rate to Prescribe Physical Activity: Cross-Sectional Exploratory Study

**DOI:** 10.2196/57373

**Published:** 2024-12-11

**Authors:** Fernanda Laís Loro, Riane Martins, Janaína Barcellos Ferreira, Cintia Laura Pereira de Araujo, Lucio Rene Prade, Cristiano Bonato Both, Jéferson Campos Nobre Nobre, Mariane Borba Monteiro, Pedro Dal Lago

**Affiliations:** 1 Graduate Program of Rehabilitation Sciences Universidade Federal de Ciências da Saúde de Porto Alegre - UFCSPA Porto Alegre Brazil; 2 Undergraduate Course of Medicine Universidade Federal de Ciências da Saúde de Porto Alegre Porto Alegre Brazil; 3 Department of Physical Therapy Universidade Federal de Ciências da Saúde de Porto Alegre - UFCSPA Porto Alegre Brazil; 4 Graduate Program in Computing Sciences Universidade do Vale do Rio do Sinos - UNISINOS Porto Alegre Brazil; 5 Institute of Informatics Universidade Federal do Rio Grande do Sul - UFRGS Porto Alegre Brazil

**Keywords:** heart rate, wearable device, HR, biosensor, physiological monitor, wearable system, medical device, mobile phone

## Abstract

**Background:**

Wearable sensors are rapidly evolving, particularly in health care, due to their ability to facilitate continuous or on-demand physiological monitoring.

**Objective:**

This study aimed to design and validate a wearable sensor prototype incorporating photoplethysmography (PPG) and long-range wide area network technology for heart rate (HR) measurement during a functional test.

**Methods:**

We conducted a transversal exploratory study involving 20 healthy participants aged between 20 and 30 years without contraindications for physical exercise. Initially, our laboratory developed a pulse wearable sensor prototype for HR monitoring. Following this, the participants were instructed to perform the Incremental Shuttle Walk Test while wearing the Polar H10 HR chest strap sensor (the reference for HR measurement) and the wearable sensor. This test allowed for real-time comparison of HR responses between the 2 devices. Agreement between these measurements was determined using the intraclass correlation coefficient (ICC_3.1_) and Lin concordance correlation coefficient. The mean absolute percentage error was calculated to evaluate reliability or validity. Cohen *d* was used to calculate the agreement’s effect size.

**Results:**

The mean differences between the Polar H10 and the wearable sensor during the test were –2.6 (95% CI –3.5 to –1.8) for rest HR, –4.1 (95% CI –5.3 to –3) for maximum HR, –2.4 (95% CI –3.5 to –1.4) for mean test HR, and –2.5 (95% CI –3.6 to –1.5) for mean recovery HR. The mean absolute percentage errors were –3% for rest HR, –2.2% for maximum HR, –1.8% for mean test HR, and –1.6% for recovery HR. Excellent agreement was observed between the Polar H10 and the wearable sensor for rest HR (ICC_3.1_=0.96), mean test HR (ICC_3.1_=0.92), and mean recovery HR (ICC_3.1_=0.96). The agreement for maximum HR (ICC_3.1_=0.78) was considered good. By the Lin concordance correlation coefficient, the agreement was found to be substantial for rest HR (*r*_c_=0.96) and recovery HR (*r*_c_=0.96), moderate for mean test HR (*r*_c_=0.92), and poor for maximum HR (*r*_c_=0.78). The power of agreement between the Polar H10 and the wearable sensor prototype was large for baseline HR (Cohen *d*=0.97), maximum HR (Cohen *d*=1.18), and mean recovery HR (Cohen *d*=0.8) and medium for mean test HR (Cohen *d*= 0.76).

**Conclusions:**

The pulse-wearable sensor prototype tested in this study proves to be a valid tool for monitoring HR at rest, during functional tests, and during recovery compared with the Polar H10 reference device used in the laboratory setting.

## Introduction

Recent decades have seen remarkable advancements in wearable sensor technology, a vital link between human physiological systems and wireless communication platforms [1]. This integration has led to significant innovations in biosensors and wearable sensors, particularly in the health field, enabling continuous and intermittent monitoring of physiological parameters [2].

These advancements offer a wide range of capabilities, including the detection of movement, assessment of heart rate (HR) variability, monitoring of sleep cycles, measurement of stress markers, evaluation of gait and balance, and detection of falls, as well as assessment of cutaneous temperature and respiratory parameters [3-9]. Among these metrics, HR is a critical parameter, serving as a key determinant for individualized aerobic exercise regimens across various intensity levels [10,11]. Wearable sensors, which can noninvasively capture and provide biofeedback, hold promise for optimizing exercise routines based on HR metrics [12].

The current market is saturated with numerous brands of HR monitoring devices, each claiming precision in their measurements. Popular brands include Apple Watch, Fitbit (Google), Polar (Polar Electro Oy), Xiaomi (Mi), and Garmin (Garmin Ltd) [13-15]. In this context, photoplethysmography (PPG) is the most used technology for measuring and monitoring HR [16]. Despite their widespread availability, many of these devices come with a high price tag, limiting their accessibility to a significant portion of the population. One device that has received validation for its accuracy in HR assessment is the Polar H10 chest strap, particularly when compared with the electrocardiogram, a gold-standard apparatus for HR assessment. The Polar H10 consistently demonstrates reliability during rest and various physical activity levels [17,18]. However, the cost varies from US $99.95 to US $600, representing a barrier to widespread adoption [19-21].

Existing wearable sensors on the market are often limited to connecting with their brand-specific mobile apps, which typically display HR data but lack gamification features. Gamification refers to integrating game-like elements, such as rewards, challenges, leaderboards, and feedback systems, into nongame contexts like health and fitness. These techniques have demonstrated an ability to increase user motivation, engagement, and adherence to physical activity routines by making the process more interactive and rewarding. Notably, the effects of gamification are not solely short-lived or due to novelty; research has shown that gamification can maintain long-term behavioral change by reinforcing positive habits and fostering user autonomy and competence [22,23]. Despite these benefits, a notable gap exists in integrating wearable sensors and gamification. According to a recent systematic review, combining wearable devices with gamified apps presents a promising strategy to enhance the effectiveness of interventions aimed at increasing physical activity. However, the current body of research lacks high-quality studies examining how this integration can specifically promote maintained physical activity levels [24].

In light of these considerations, this study aims to design and validate a wearable sensor prototype equipped with PPG and long-range wide area network (LoRaWAN) technology for measuring HR during functional evaluations. The ultimate goal is to incorporate this technology into a gamified app, which will provide exercise prescriptions and motivate adherence through interactive and engaging features.

## Methods

### Study Design

This transversal exploratory study was conducted from August to December 2022 at the Universidade Federal de Ciências da Saúde de Porto Alegre to evaluate the feasibility and initial outcomes of the wearable sensor prototype. The transversal design was chosen to provide a snapshot of the prototype’s performance within a specific time frame. The participants were recruited according to predefined inclusion criteria, which typically included healthy individuals aged between 20 and 30 years without physical exercise contraindications. Exclusion criteria, if any, were also clearly delineated to ensure the safety and integrity of the study participants (Multimedia Appendix 1).

### Participants

The study included healthy adults aged between 20 and 30 years without medical contraindications for physical exercise. Exclusion criteria comprised individuals with visual impairments, those with chronic conditions such as musculoskeletal or neurological diseases that could impede participation in the exercise protocol, and those who could not read or write. Since the study is an exploratory evaluation of a wearable sensor prototype, participants were recruited through convenience sampling using social media platforms, specifically WhatsApp (Meta). Invitations were distributed through various social groups and individual contacts on the platform, targeting individuals who met the predefined inclusion criteria. Before accepting the invitation, potential participants were informed about the study’s objectives, procedures, and eligibility requirements. Only those who responded positively and met the inclusion criteria were enrolled in the study, totaling 20 participants. This sample size was chosen to provide sufficient data to assess the feasibility and initial outcomes of the prototype within the constraints of the study’s scope and resources.

### Developed Wearable Sensor Prototype

The prototype operates based on HR measurement using PPG. The peak-to-peak interval of the PPG signal is used to detect the HR. However, motion artifacts can contaminate the PPG signal during physical activity, interfering with HR estimation. These artifacts primarily result from ambient movement. Some filters were applied to address this issue.

**Figure 1:**
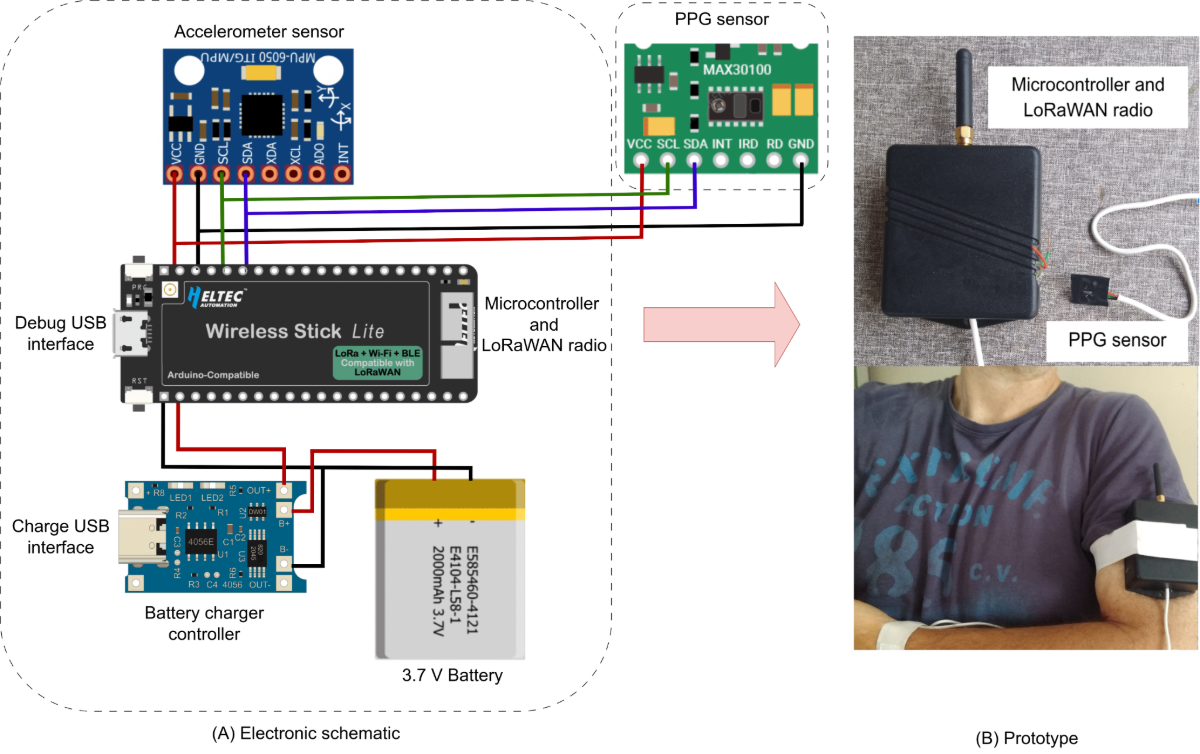
Wearable sensor prototype schematic. LoRaWAN: long-range wide area network; PPG: photoplethysmography; USB: universal serial bus.

The prototype uses PPG technology and incorporates a traditional PPG HR extraction algorithm based on the discrete Fourier transform [25]. PPG is a method that measures changes in light absorption corresponding to arterial blood volume fluctuations during systole using optical measurements [26,27]. The peak-to-peak interval of the PPG signal is used to detect the HR. Similar to the technology used in pulse oximeters, the sensor relies on optical techniques to estimate HR [26,28]. It consists of a photodetector and a light source that illuminates the skin to detect variations in light caused by changes in skin blood flow [28]. However, motion artifacts can harm the PPG signal during physical activity, interfering with HR measurement. These artifacts primarily result from ambient movement. Some filters were applied to address this issue. The collected data were stored in the cloud. Following prototype development, the LoRaWAN network was selected for transmitting sensor data over an exercise area of up to 10 km. After preprocessing, the sensor data are transmitted through LoRaWAN to a central concentrator, where they are encrypted and subsequently sent to a secure cloud-based database. Each user’s data history is stored in this cloud database, allowing for sensor validation, statistical analysis, and future integration with gamification interfaces. Data were continuously collected at a sample rate of 500 Hz and securely stored in the cloud, capturing detailed information with each heartbeat. Figure 1 illustrates the schematic diagram of the wearable sensor prototype, including its circuit, and provides a photo of the physical prototype. The prototype weighs approximately 150 grams and measures 5 × 7 centimeters.

### Data Collection

**Figure 2:**
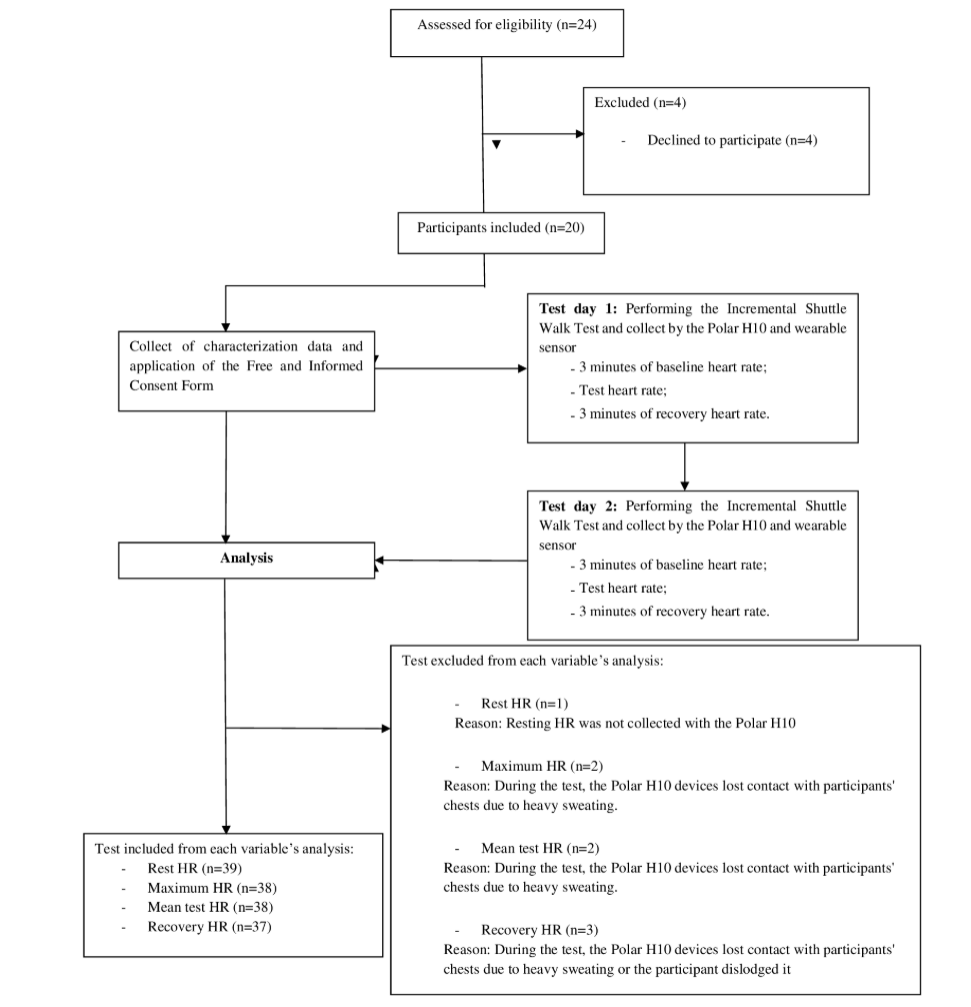
Flowchart of inclusion and methodical procedure. HR: heart rate.

Following the development of the wearable sensor prototype for HR monitoring, a validation test was conducted using the Incremental Shuttle Walk Test (ISWT), a well-established and safe walking test for assessing functional capacity [28,29]. The participants underwent 2 ISWTs, with the second test administered 7 to 14 days after the initial examination. Figure 2 presents the flowchart of inclusion and systematic procedures.

The ISWT involves graded bidirectional movement along a 10-meter corridor in response to audio cues. The standard test consists of 12 one-minute stages, starting at 0.5 m/s and increasing by 0.17 m/s each minute [29,30]. This study modified the protocol to include 3 additional stages, potentially allowing healthy participants to reach maximum exertion [31]. The test concludes when participants signal their inability to continue or fail to maintain the pace [30]. During the ISWT, participants wore the wearable sensor prototype on their nondominant wrist and the Polar H10 chest strap (Polar Electro Oy), the HR reference [17,18]. HR data were collected at three points: (1) at three minutes pretest, (2) during the test, and (3) at three minutes posttest. The average HR collected during each period was used in the analyses.

### Statistical Analysis

The quantitative outcomes were depicted as means and SD. The normality of the data was assessed using the Shapiro-Wilk test.

The 2-tailed *t* test was used to compare HR measurements from the Polar H10 and the wearable sensor prototype. Agreement between these measurements was determined using the intraclass correlation coefficient (ICC_3,1_), with interpretations categorized as (1) <0.5=poor; (2) 0.5-0.75=moderate; (3) 0.75-0.9=good; and (4) >0.9=excellent [32]. In addition, the Lin concordance correlation coefficient was used to assess agreement between methods, with interpretations of (1) <0.9=poor; (2) 0.9-0.95=moderate; (3) 0.95-0.99=substantial; (4) >0.99=almost perfect [33].

The mean absolute percentage error (MAPE) was calculated to evaluate reliability or validity, with MAPE values ≤5% indicating high reliability or validity [34]. To calculate the effect size of agreement, Cohen *d* was used, with interpretations of (1) ≥1=very large; (2) 0.8=large; (3) 0.5=medium; (4) 0.2=small [35]. Bland-Altman plots were used to display agreement upper and lower limits and bias (mean difference), following the approach described by Bland and Altman [36]. A significance level of .05 was set for all tests. All analyses were conducted using IBM SPSS Statistics for Windows (version 27.0; IBM Corp.).

### Ethical Considerations

The study conformed to the resolution 466/2012 of the Brazilian National Health Council. It was approved by the local ethical committee for Research on Human Beings at the Universidade Federal de Ciências da Saúde de Porto Alegre (approval 54492221.80000.5345). Before participation, all individuals provided informed consent by signing the Informed Consent Form. No financial incentives were given to participants in the research.

## Results

A total of 20 participants were recruited, consisting of 12 men and 8 women, with a mean age of 23.3 (SD 2.1) years, height of 169 (SD 8.4) cm, weight of 71 (SD 31) kg, and BMI of 24.5 (SD 4.5) kg/m². The distance performed in the ISWT was 1190.2 (SD 250.6) m.

The Shapiro-Wilk test was used to test the normality of Polar H10 baseline HR (*P*=.02), Polar H10 maximum HR (*P*=.08), Polar H10 mean test HR (*P*=.88), Polar H10 recovery HR (*P*=.04), prototype baseline HR (*P*=.18), prototype maximum HR (*P*=.26), prototype mean test HR (*P*=.08), prototype recovery HR (*P*=.05).

Comparison of HR measurements between the Polar H10 and the wearable sensor prototype revealed the following mean differences (Table 1): (1) rest HR –2.6 (95% CI –3.5 to –1.8); (2) maximum HR –4.1 (95% CI –5.3 to –3); (3) mean test HR –2.4 (95% CI –3.5 to –1.4); and (4) mean recovery HR –2.5 (95% CI –3.6 to –1.5).

**Table 1:** Mean, SD, and mean absolute percentage error obtained for heart rate (HR) of the chest strap of the Polar H10 sensor and wearable sensor prototype during the rest period, maximum HR, mean test HR, and mean recovery HR (after 3 minutes).

	Polar H10, mean (SD)	Prototype, mean (SD)	Difference (Polar H1-prototype)		
			Bias	MAPE%^a^	95% CI
Rest HR (bpm^b^)	86.7 (12.6)	89.4 (12.5)	–2.6	–3	–3.5 to –1.8
Maximum test HR (bpm)	190.7 (7)	194.9 (8.4)	–4.1	–2.2	–5.3 to –3
Mean test HR (bpm)	137.5 (9.7)	139.9 (9.7)	–2.4	–1.8	–3.5 to –1.4
Recovery HR (bpm)	153.8 (13.6)	156.3 (14.2)	–2.5	–1.6	–3.6 to –1.5

^a^MAPE: mean absolute percentage error.

^b^bpm: beats per minute.

Analyses demonstrated excellent agreement between the Polar H10 chest strap and the wearable sensor prototype (Table 2) for rest HR (ICC_3.1_=0.96), mean test HR (ICC_3.1_=0.92), and mean recovery HR (ICC_3.1_=0.96) and good agreement for maximum HR (ICC_3.1_=0.78). By the Lin concordance correlation coefficient, the agreement was found to be substantial for rest HR (*r*_c_=0.96) and recovery HR (*r*_c_=0.96), moderate for mean test HR (*r*_c_=0.92), and poor for maximum HR (*r*_c_=0.78). The power of agreement between Polar H10 and the wearable sensor prototype was large for baseline HR, maximum HR, and mean recovery HR and medium for mean test HR (Table 2).

**Table 2:** Agreement between the heart rate measurement of the wearable sensor prototype and Polar H10 evaluated by the intraclass correlation coefficient (ICC3.1), Lin concordance correlation coefficient (rc), and effect size of agreement (Cohen *d*).

	ICC_3.1_	95% CI	*r*_c_	95% CI	Cohen *d*
Rest HR^a^	0.96	0.71-0.98	0.95	0.92-0.97	0.97
Maximum test HR	0.78	0.09-0.93	0.78	0.66-0.86	1.18
Mean test HR	0.92	0.7-0.97	0.92	0.85-0.95	0.76
Recovery HR	0.96	0.82-0.98	0.96	0.92-0.97	0.8

^a^HR: heart rate.

**Figure 3:**
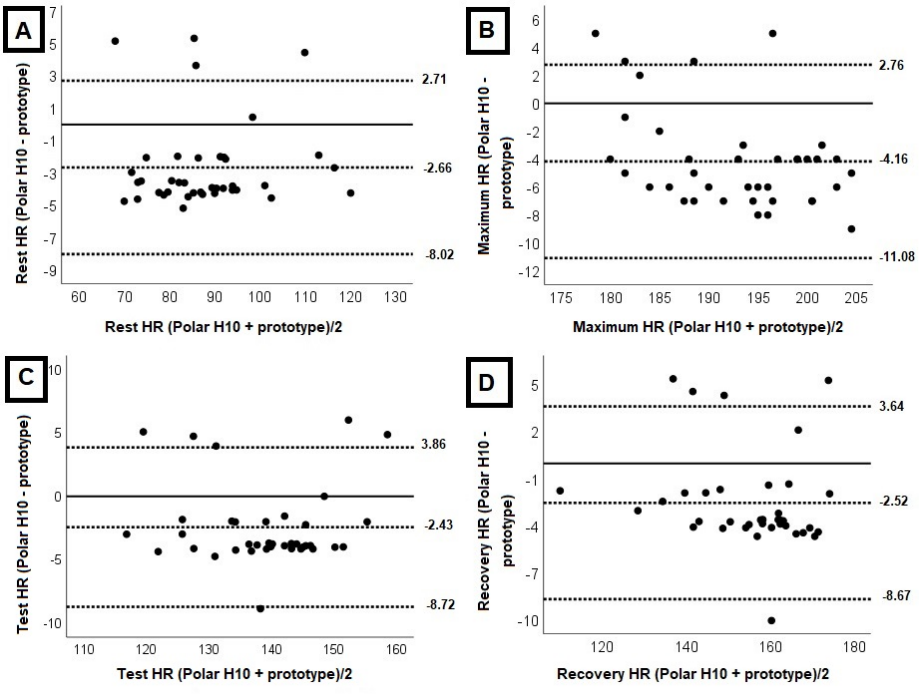
Bland-Altman analysis comparing the Polar H10 sensor chest strap and the wearable sensor prototype on (A) heart rate (HR) during the rest period, (B) maximum HR, (C) mean test HR (C), and (D) recovery HR. The center solid line in each plot represents the mean bias (difference) between each paired value as absolute HR. The top and bottom dashed lines are 1.96 SDs from the mean difference.

Bland-Altman [36] plots (Figure 3) depict the agreement for all variables. Most HR measurements at rest, during the test, and recovery fell within the upper and lower limits of the Bland-Altman [36] graphs, indicating measurement agreement. Although some tests did not fall within these limits, the error is tolerable, as the values are not clinically significant for exercise prescription based on HR zones.

## Discussion

### Principal Findings

Our analysis revealed a significant agreement between the HR measurements taken by our developed wearable sensor prototype and the Polar H10 strap. The wearable sensor is a valid instrument for HR monitoring during rest, exercise, and recovery periods. However, the prototype does not accurately measure maximum HR.

### Comparison to Previous Work

Low-cost sensors (priced below US $100) are pivotal in clinical practice and rehabilitation. They aid in physiological measurements for diagnosis and evidence-based practices and are vital for gamified apps promoting physical activity and rehabilitation [37]. Our findings advance the incorporation of low-cost sensor feedback into gamified apps.

We opted for LoRaWAN technology due to its cost-effectiveness and expansive range. Furthermore, the ease of its integration with smartphones made it a compelling choice [38].

Monitoring HR is pivotal for accurate exercise intensity determination and prescription. It is a proven method for athletes, healthy individuals, and patients with cardiovascular conditions [10,11,39-42]. Our results corroborate that our wearable sensor is reliable, with excellent ICCs to rest HR, mean test HR, and recovery. According to the Lin concordance correlation coefficient, the agreement ranges from substantial to moderate in the same variables [33].

Our error percentages are in alignment with the established literature. For instance, Fuller et al [13] showcased that 56.5% of wearable HR measurements had an error within 3%. Our prototype displayed consistent accuracy with MAPE values lower than 3%, aligning with previous research stipulating such thresholds for reliability and validity [13].

We observed the most significant MAPE during resting periods. However, as the literature suggests, pulse sensors often exhibit reduced MAPE during escalated speeds [15,42]. This behavior matches our findings during the ISWT.

The agreement was within the Bland-Altman plots’ upper and lower limits [36], but some tests were outside this range. This outside may be due to the difference in the technology used by the Polar H10 and the prototype that used the PPG. In this case, PPG technology has a limitation of susceptibility to motion artifacts caused by hand movement and differences in photosensitivity between individuals, which may limit data precision [43,44]. In the Bland-Altman plots [36], the agreement must be evaluated from a clinical point of view. In this context, the error HR is tolerable and does not limit the use of the wearable sensor prototype in exercise.

### Strengths and Limitations

Our study has some potential limitations: (1) it was conducted in a controlled laboratory environment, which may not fully capture real-world conditions; (2) participants were healthy and belonged to a younger age group, limiting the generalizability of our findings to broader populations; (3) temperature conditions were controlled between 20 and 24°C, and we did not explore how the device performs under varying environmental conditions, which could impact its reliability; and (4) we did not compare the wearable sensor with others across different temperatures. These factors limit the applicability of our findings to diverse settings, age groups, and health statuses. Future studies will address these limitations by testing the wearable sensor in uncontrolled environments and across a broader range of populations and conditions, including older adults and individuals with varying health statuses. In addition, further investigation is needed to assess how different environmental factors, such as temperature, affect sensor performance.

One of the key strengths of our study is the creation of a low-cost wearable sensor that can be integrated into a gamified app. This approach not only makes the technology accessible but also encourages user engagement through interactive features. The wearable’s accurate HR measurement ensures safe exercise intensity recommendations, making it a valuable tool for personalized fitness monitoring.

### Future Directions

Our device is precisely engineered to seamlessly integrate with gamified apps, enhancing user experience through real-time HR biofeedback. The traditional devices are often limited to brand-specific apps, our prototype leverages an open database architecture that allows for flexibility and interoperability with various platforms. It transmits HR signals through an antenna and stores them in a cloud-based database, enabling real-time access and processing of HR data. It is essential to effectively implement gamification strategies. Key features that make our device particularly suitable for this integration include the following. First, real-time HR biofeedback: the ability to transmit HR data in real time is crucial for interactive gamified experiences, allowing users to receive instant feedback on their performance and adjust their activity accordingly. Second, open database architecture: the flexibility of our open architecture enables compatibility with various fitness and gamification platforms, supporting the customization of exercise routines, challenges, and user profiles based on real-time data. This flexibility allows third-party developers to easily integrate the device into their systems. Third, cloud-based scalability: the cloud storage system provides secure and scalable data management and supports advanced analytics. This enables integration with machine learning algorithms that dynamically adjust game elements (eg, difficulty levels, rewards) based on user performance and trends. Fourth, personalization and adaptability: our device offers personalized feedback by analyzing the user’s HR data, making it highly adaptive to individual needs. This enhances engagement by offering customized rewards, progress tracking, and social interaction—proven elements of effective gamification. These features empower users by fostering a more interactive and responsive fitness ecosystem. The combination of real-time data processing, personalized feedback, and cloud-based analytics uniquely equip the device to enhance motivation and adherence to health goals through gamification.

This study is a pilot, and we plan to conduct further research to refine both the wearable sensor and the algorithm used for HR measurement, specifically using the discrete Fourier transform to improve accuracy, particularly when measuring maximum HR. The prototype does not consistently measure maximum HR with optimal precision. This may be due to 2 primary factors: further refinement of the PPG algorithm and motion artifacts during high-intensity exercise. To address these limitations, future improvements will focus on enhancing the PPG algorithm to filter out noise better and more accurately track HR at higher intensities. In addition, we plan to implement techniques to mitigate motion artifacts, such as using more advanced filtering methods or improving the sensor’s attachment to the body to reduce movement interference. These enhancements are expected to improve the sensor’s overall performance and accuracy in measuring maximum HR.

After refining the algorithm, we also plan to reduce the device’s weight and size, addressing other ergonomic issues. We will use 3D printing technology to help refine the design and reduce the size. This iteration will enhance ergonomic comfort and make the device more convenient and practical for users during physical exercise.

The production cost of the wearable sensor prototype was approximately US $38.50, excluding the antenna. At this stage, our objective is not to conduct a cost-effectiveness study, as the wearable sensor prototype is still under development. Therefore, we cannot present a final price or provide a detailed cost comparison with other commercially available devices. In future research, we plan to perform a cost-effectiveness study and compare our prototype’s costs and capabilities with similar devices.

### Conclusion

In conclusion, our wearable sensor prototype effectively measures HR, drawing parallels with the Polar H10 sensor for rest HR during testing and recovery in the laboratory environment. Future work will involve integrating this wearable sensor prototype into gamified apps based on the validation performed in this work. This integration is expected to enhance adherence to regular exercise and ensure accurate intensity prescription, thereby maximizing the potential benefits for users.

## Appendix

Multimedia Appendix 1 STROBE (Strengthening the Reporting of Observational Studies in Epidemiology) statement.

## Abbreviations

HR: heart rate
ICC: intraclass correlation coefficient
ISWT: Incremental Shuttle Walk Test
LoRaWAN: long-range wide area network
MAPE: mean absolute percentage error
PPG: photoplethysmography

## Data Availability

The datasets generated during and/or analyzed during this study are available from the corresponding author upon reasonable request.
